# Detection of Pulmonary Embolism in Returning Travelers with Hypoxemic Pneumonia due to COVID-19 in Reunion Island

**DOI:** 10.4269/ajtmh.20-0597

**Published:** 2020-07-01

**Authors:** Kevin Larsen, Nathalie Coolen-Allou, Laurie Masse, Alexandre Angelino, Jérôme Allyn, Lea Bruneau, Adrien Maillot, Marie Lagrange-Xelot, Thierry Vitry, Michel André, Jean Yves Travers, Emilie Foch, Nicolas Allou

**Affiliations:** 1 Pneumologie, Centre Hospitalier Universitaire Felix Guyon Allée des Topazes, Saint Denis, France;; 2 Radiologie, Centre Hospitalier Universitaire Felix Guyon Allée des Topazes, Saint Denis, France;; 3 Réanimation Polyvalente, Centre Hospitalier Universitaire Felix Guyon Allée des Topazes, Saint Denis, France;; 4 INSERM CIC 1410 Clinical and Epidemiology, University Hospital, Saint Denis, France;; 5 Department of Public Health and Research Support, Methodological Support and Biostatistics Unit, University Hospital, Saint Denis, France;; 6 Service des Maladies Infectieuses, Centre Hospitalier Universitaire Felix Guyon Allée des Topazes, Saint Denis, France

## Abstract

The aim of this study was to evaluate the occurrence of pulmonary embolism in returning travelers with hypoxemic pneumonia due to COVID-19. All returning travelers to Reunion Island with hypoxemic pneumonia due to COVID-19 underwent computed tomography pulmonary angiography (CTPA) and were included in the cohort. Thirty-five patients were returning travelers with hypoxemic pneumonia due to COVID-19 and had recently returned from one of the countries most affected by the COVID-19 outbreak (mainly from France and Comoros archipelago). Five patients (14.3%) were found to have pulmonary embolism and two (5.9%) were incidentally found to have deep vein thrombosis on CTPA. Patients with pulmonary embolism or deep vein thrombosis had higher D-dimer levels than those without pulmonary embolism or deep vein thrombosis (*P* = 0.04). Returning travelers with hypoxemic pneumonia due to COVID-19 should be systematically screened for pulmonary embolism.

An outbreak of COVID-19 that started in China in December 2019 began to spread globally,^1^ particularly in Europe, in January 2020. Reunion Island (845,000 inhabitants) is a French overseas department located in the Indian Ocean. It is at a distance of 10,000 km from Paris and is connected to metropolitan France by several daily flights (11 hours).

Between March 11, 2020 and April 20, 2020, 388 cases of COVID-19 were reported in Reunion Island.^2^ The COVID-19 pandemic is overwhelmingly associated with international travel.^3^ Long-haul flight travel is a known risk factor of pulmonary embolism.^4^ The aim of this study was to evaluate the occurrence of pulmonary embolism in returning travelers with hypoxemic pneumonia due to COVID-19.

The present observational study was approved by the French Ethics Committee of Infectious Disease and Tropical Medicine and was declared to the Commission nationale de l’informatique et des libertés (French Data Protection Agency or CNIL, N° 2206739).

This observational study was conducted between March 11, 2020 (first case of COVID-19 in Reunion Island) and April 20, 2020 at Félix Guyon University Hospital, the only hospital authorized to handle patients with COVID-19 in Reunion Island. In accordance with our protocol, all patients with hypoxemic pneumonia due to COVID-19 confirmed by polymerase chain reaction underwent systematic chest computed tomography angiography pulmonary (CTPA). Among patients with hypoxemic pneumonia, all returning travelers were included in the cohort study. Hypoxemic pneumonia was defined as pneumonia requiring oxygen supplementation to achieve oxyhemoglobin saturation > 94%. All CTPA images were analyzed by two radiologists (T. V. and J. Y. T.) and at least two pulmonologists (L. M., E. F., M. A., and N. C. A.) blinded to clinical information. In cases of CTPA contraindications such as contrast allergy or renal failure, patients underwent lung ventilation/perfusion scintigraphy instead. Computed tomography angiography pulmonary examinations were performed in multi-detector computed tomography scanners (GE Revolution GSI, General Electric, Milwaukee, WI) by using a standard CTPA protocol. Scan parameters were as follows: tube voltage of 100 kV, tube current of 100–300 mAs, collimation of 0.625 mm, pitch of 1.375–1.0, table speed of 55 mm/second, and gantry rotation time of 0.5 seconds. Images were reconstructed with a thickness of 1 mm and an increment of 1.25 mm.

Results were expressed as total numbers (percentages) for categorical variables and as medians (25th–75th percentiles) for continuous variables. Categorical variables were compared using the chi-square test or the Fisher’s exact test, as appropriate. A *P-*value < 0.05 was considered significant. Analyses were performed using SAS statistical software (8.2, Cary, NC).

Over the study period, 165 of 388 patients (42.5%) who had tested positive for COVID-19 were admitted to Félix Guyon University Hospital. Of these 165 patients, 44 (26.7%) patients had hypoxemic pneumonia.

Thromboembolic events occurred in seven of the 35 (20%) returning travelers versus one of the nine (11%) non-returning travelers (*P* = 0.35). The 35 returning travelers had recently returned from one of the countries most affected by the COVID-19 outbreak: 24 from metropolitan France, eight from Comoros archipelago, five from Spain, two from Italy, two from the United States, and one from the United Kingdom (some patients had visited several of these countries). Patient characteristics at study inclusion are shown in Table 1. The median duration between onset of symptoms and diagnosis of COVID pneumonia was 6 (4–9) days. The median number of days between arrival to Reunion Island and the first day of symptoms was 0 (-3–2) day.

**Table 1:** Characteristics of the 45 patients

Characteristic	Total (*n* = 35)	Patients with PE or extremity deep vein thrombosis		*P*-value
		No (*n* = 28)	Yes (*n* = 7)	
Quick sequential organ failure assessment score	1 (0–1)	1 (0–1)	1 (1–1)	0.46
Male gender, *n* (%)	27 (77.1)	20 (71.4)	7 (100)	0.17
Age (years)	66 (56–78)	69 (59–80)	50 (56–68)	0.2
Hypertension, *n* (%)	15 (42.9)	12 (42.9)	3 (42.9)	1
Diabetes mellitus, *n* (%)	6 (17.1)	5 (17.9)	1 (14.3)	1
Body mass index > 30 kg/m^2^, *n* (%)	5 (14.3)	4 (14.3)	1 (14.3)	1
Chronic kidney disease, *n* (%)	3 (8.6)	3 (10.7)	0	1
History of DVT or PE, *n* (%)	3 (8.6)	2 (7.1)	1 (14.3)	1
Chronic obstructive pulmonary disease, *n* (%)	6 (17.1)	5 (17.9)	1 (14.3)	1
History of congestive heart failure, *n* (%)	9 (25.7)	8 (28.6)	1 (14.3)	0.65
Cancer (< 3 months), *n* (%)	4 (11.4)	2 (7.1)	2 (28.6)	0.17
Tobacco smoking, *n* (%)	2 (5.7)	2 (7.1)	0	1
Dyspnea, *n* (%)	21 (60)	16 (57.1)	5 (71.4)	0.68
Chest pain, *n* (%)	5 (14.3)	4 (14.3)	1 (14.3)	1
Right bundle branch block or S1Q3, *n* (%)	1 (2.9)	1 (3.6)	0	1
Leukocyte count (G/L)	6.46 (4.5–10.2)	5.46 (4.22–9.45)	7.28 (6.2–17.8)	0.07
Lymphocytes count (G/L)	1.17 (0.79–1.36)	1.17 (0.84–1.34)	0.93 (0.56–1.49)	0.478
D-dimer level (µg/mL)	1.22 (0.63–3.19)	0.99 (0.62–1.79)	3.01 (1.48–11.30)	0.04
C-reactive protein (mg/dL)	76.8 (23.9–130)	72 (23–115)	155 (61–230)	0.12
Cardiac troponin I > 10 ng/L, *n* (%)	12 (34.3)	8 (28.6)	4 (57.1)	0.16
Lactate dehydrogenase (IU/L)	393 (287–464)	376 (285–450)	436 (331–517)	0.29
Creatinine level (µmol/L)	90 (76–106)	90 (72–105)	107 (88–169)	0.12
Low molecular weight heparin prophylaxis, *n* (%)	28 (80)	25 (89.3)	3 (42.9)	0.02
Bilateral involvment on CT scan, *n* (%)	31 (88.6)	24 (85.7)	7 (100)	0.56
Extension of pulmonary infiltrates > 50% on CT scan, *n* (%)	18 (51.4)	14 (50)	4 (57.1)	1
Pleural effusion on CT scan, *n* (%)	9 (25.7)	6 (21.4)	3 (42.9)	0.34

CT = computed tomography; NS = nonsignificant; PE = pulmonary embolism. Results are expressed at *n* (%) or median (25th–75th) as appropriate.

Of the 35 enrolled patients, 33 (94.3%) underwent one CTPA, one (2.9%) underwent two CTPAs, and one (2.9%) underwent one lung ventilation/perfusion scintigraphy. Chest examinations were performed on day 7 (5–12) after the onset of symptoms, and the median value of oxygen therapy was 4 (2–13) L/minutes. Four of the 35 patients (11.4%) received invasive mechanical ventilation.

Five patients were found to have pulmonary embolism (14.3%). Among the five patients, one had an associated aortic arch thrombosis (Figure 1). Two other patients were (incidentally) found to have extremity deep vein thrombosis (extensive catheter-related jugular thrombosis) on CTPA, for a total of minimum of 20% of the enrolled patients.

**Figure 1.**
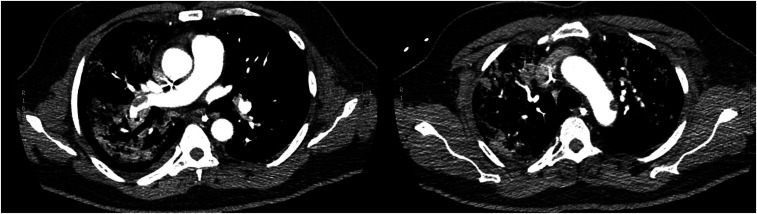
Axial computed tomography pulmonary angiography performed on day 17 after the onset of symptoms. This patient presented with thrombi in both branches of pulmonary artery and aortic arch thrombosis.

After univariate analysis, the factors associated with pulmonary embolism and extremity deep vein thrombosis were higher levels of D-dimer (*P* = 0.04) and absence of low molecular weight heparin prophylaxis (*P* = 0.02) (Table 1). There were no deaths at follow-up (minimum 40 days, and only two remained hospitalized and had been weaned from oxygen therapy).

To our knowledge, this is the only study that has consecutively evaluated the occurrence of pulmonary embolism in returning travelers with hypoxemic pneumonia due to COVID-19. In our study, the incidence of thromboembolic complications was high despite the fact that the patients had a low severity score with a quick sepsis-related organ failure assessment score of 1 (0–1) (97.1% had a severity score < 2).

In the published literature, the incidence of thrombotic complications like pulmonary embolism or deep vein thrombosis in patients with COVID-19 pneumonia is highly variable, ranging from 1% to 20%.^5–7^ The incidence of thrombotic complications in our study population may seem relatively high despite that severity score was very low. The incidence that was reported in the retrospective study by Chen et al.^6^ was lower (< 1%)—though it should be noted that only 25 of the 1,008 patients (2.5%) examined in that study had undergone CTPA. In the other studies published on the topic, the incidence of thrombotic complications was high because only patients hospitalized in intensive care were evaluated.^7,8^ Deep vein thrombosis and pulmonary embolism are severe complications of COVID-19. It is important that they can be diagnosed early because delay in treatment can be life-threatening in the short and long term. In our study, patients without pulmonary embolism had a median D-dimer level < 1 µg/mL. In the study by Chen et al.,^6^ the median D-dimer level of patients without pulmonary embolism was 2.44 µg/mL. In the study by Cui et al.,^7^ a level of D-dimer of 1.5 µg/mL was a good cutoff to predict venous thromboembolism (sensitivity of 85.0% and specificity of 88.5%). Last, retrospective studies suggest that preventive anticoagulation is associated with decreased mortality in patients with severe COVID-19 infection, particularly in those with high D-dimer levels.^8^ Enhanced preventive anticoagulation should be used in all patients with hypoxemic pneumonia due to COVID-19.^9^

The main limitations of our study are that the number of evaluated patients and the number of events were relatively small. Moreover, it was not possible to perform a control group because the vast majority of COVID-19 patients came from Europe by long-haul flight (i.e., > 6,000 km). Reunion Island is located at a distance of 10,000 km from Europe. Air flight is a well-known risk factor for the occurrence of pulmonary embolism, and this risk increases with the duration of the flight.^10^

In conclusion, the incidence of pulmonary embolism and deep vein thrombosis in our study population was relatively high at 20%. Returning travelers with hypoxemic pneumonia due to COVID-19 should be systematically screened for pulmonary embolism or deep vein thrombosis regardless of the level of D-dimers.
